# Advanced hybrid LSTM-transformer architecture for real-time multi-task prediction in engineering systems

**DOI:** 10.1038/s41598-024-55483-x

**Published:** 2024-02-28

**Authors:** Kangjie Cao, Ting Zhang, Jueqiao Huang

**Affiliations:** 1 Key Laboratory of Ethnic Language Intelligent Analysis and Security Governance of MOE, Minzu University of China, No. 27 Zhongguancun South Avenue, Beijing, 100081 China; 2 Present Address: School of Information and Engineering, Minzu University of China, Beijing, 100081 China

**Keywords:** LSTM-transformer hybrid architecture, Real-time predictions, Engineering systems, Underground drilling, Green stormwater management, Multi-task learning, Online learning, Knowledge distillation, Predictive accuracy, Computer science, Engineering, Computational science, Scientific data

## Abstract

In the field of engineering systems—particularly in underground drilling and green stormwater management—real-time predictions are vital for enhancing operational performance, ensuring safety, and increasing efficiency. Addressing this niche, our study introduces a novel LSTM-transformer hybrid architecture, uniquely specialized for multi-task real-time predictions. Building on advancements in attention mechanisms and sequence modeling, our model integrates the core strengths of LSTM and Transformer architectures, offering a superior alternative to traditional predictive models. Further enriched with online learning, our architecture dynamically adapts to variable operational conditions and continuously incorporates new field data. Utilizing knowledge distillation techniques, we efficiently transfer insights from larger, pretrained networks, thereby achieving high predictive accuracy without sacrificing computational resources. Rigorous experiments on sector-specific engineering datasets validate the robustness and effectiveness of our approach. Notably, our model exhibits clear advantages over existing methods in terms of predictive accuracy, real-time adaptability, and computational efficiency. This work contributes a pioneering predictive framework for targeted engineering applications, offering actionable insights into.

## Introduction

Modern engineering systems are increasingly becoming digitized and automated, requiring sophisticated control mechanisms to ensure their robustness and efficiency^1,2^. The integration of advanced sensors and interconnected devices contributes to the complexity of these systems^3^. In this context, real-time monitoring and predictive analytics are critical for anticipating system failures and maintaining optimal performance^4,5^.

### Background and challenges

From the dawn of industrialization to the early twenty-first century, engineering systems predominantly banked on rule-based algorithms and traditional statistical methods for their monitoring and predictive needs. While these techniques laid the foundation for system analytics, they often showed signs of strain when confronted with complex scenarios.

In complex scenarios, rule-based algorithms and traditional statistical methods may have certain limitations^6^. Rule-based algorithms often rely on domain experts to define rules and features. This can limit the scalability and adaptability of the algorithms^4^, especially in complex scenarios where domain experts' knowledge may not cover all possible cases.Traditional statistical methods often make assumptions about the data distribution, such as the assumption of normal distribution. However, in complex scenarios, the data distribution may deviate from these assumptions, leading to decreased accuracy of statistical methods. Rule-based algorithms and traditional statistical methods typically assume linear relationships between data. However, in complex scenarios, the relationships between data may be non-linear, limiting the predictive capabilities of these methods.

The current age, marked by dynamic and data-rich environments, only accentuates these strains. The Internet of Things (IoT), a revolutionary concept, has acted as a catalyst, causing an explosive surge in the volume, velocity, and variety of data generated by engineering systems^7,8^. From nuanced sensor readings capturing the minutest of vibrations to extensive operational logs detailing system workflows, the spectrum of data is vast. This mosaic of information conceals within it intricate patterns, deep-seated temporal dependencies, and rich contextual nuances, elements that often elude classical analytical methods^9^.

Additionally, the modern engineering landscape is characterized by its fluidity. Systems, environments, and requirements evolve, demanding decision-making models that are not just accurate but agile. The rigidity of traditional models, coupled with their need for periodic manual recalibration, makes them cumbersome and less effective in these dynamic environments.

Modern engineering systems require accurate predictive models to support decision-making and optimize operations. Traditional single-model approaches like LSTM and Transformer may not always meet the demand for high accuracy predictions in certain scenarios. Modern engineering systems often face dynamic operating conditions and continuously changing data streams. Traditional batch learning methods are unable to adapt in real-time to such changes, resulting in delayed or inaccurate predictions. Engineering systems often require predictions and decision-making in real-time or near real-time environments. Traditional complex models may face challenges in terms of computational efficiency, leading to slow prediction speeds or excessive computational resource consumption.Therefore, there is a need for a new hybrid architecture to overcome these challenges and meet the specific requirements of modern engineering systems.

### The promise of deep learning

At this crossroads, deep learning emerges, offering a glimmer of hope. As a more evolved subset of machine learning, deep learning ventures beyond the superficial layers of data, diving deep to extract patterns and insights. Among the arsenal of deep learning tools, Long Short-Term Memory (LSTM) networks^3^, a specialized breed of recurrent neural networks (RNNs), have carved a niche for themselves (“long short-term memory" (LSTM), a novel recurrent network architecture in conjunction with an appropriate gradient-based learning algorithm. It can learn to bridge time intervals in excess of 1000 steps even in case of noisy, incompressible input sequences, without loss of short time lag capabilities. This is achieved by ancient, gradient-based algorithm for an architecture enforcing constant error flow through internal states of special units). Their unique architectural design, replete with memory cells and meticulously crafted gates, bestows upon them the ability to recognize, capture, and retain long-term dependencies typical of time-series data. Such attributes render LSTMs an ideal candidate for a plethora of engineering applications, especially those inundated with sensor data, operational logs, and other sequential datasets^10^.

Yet, no tool is without its limitations. LSTMs, for all their prowess in sequence comprehension, occasionally exhibit vulnerabilities in situations that demand a holistic understanding of broader contextual information. (When using LSTM alone for prediction, there may be cases where the prediction results are inaccurate). This gap is addressed by the Transformer architecture^11,12^, a prodigy in the deep learning domain. Equipped with potent self-attention mechanisms, Transformers possess the innate capability to weigh the relevance of different parts of an input sequence. This discernment allows them to fathom both granular and macro-level contexts with equal finesse^13^. (However, when dealing with long sequences, Transformer faces the challenge of high computational complexity, which can hinder its effectiveness in handling long sequences. If Transformer is used alone for prediction, there may be issues of high computational complexity and slow prediction speed).

### Our contribution

In the intricate realm of engineering challenges and deep learning solutions, our research makes a substantial contribution by introducing a novel hybrid LSTM-Transformer architecture. This model is meticulously crafted to address the specific requirements of modern engineering systems, particularly in the areas of smart manufacturing and renewable energy management. Smart manufacturing and renewable energy management are highly significant fields in today's society, with implications for improving production efficiency, reducing energy consumption, and minimizing environmental impact. Therefore, researching predictive models in these two domains can provide valuable support for practical applications.Both smart manufacturing and renewable energy management face complex data and operational conditions. In smart manufacturing, there are large amounts of sensor data, optimization of production lines, and fault detection, among other challenges^2^. In renewable energy management, factors such as weather variations and energy supply–demand balance need to be considered. The complexity and challenges in these domains make researching predictive models even more meaningful and valuable^5^.

From the data and algorithmic viewpoints, smart manufacturing and renewable energy management share some commonalities and differences. Both domains involve a significant amount of time series data and sensor data. These data often exhibit high dimensionality, high frequency, and complex interdependencies. Therefore, handling these common data characteristics is a crucial challenge for predictive models^7^. Smart manufacturing and renewable energy management differ in terms of data sources and characteristics. In smart manufacturing, data primarily come from production lines, equipment, and sensors, involving production processes and quality control aspects. In renewable energy management, data mainly come from weather observations, energy production, and consumption. Therefore, designing appropriate predictive models and algorithms that account for the specific characteristics of each domain is necessary.

Unlike traditional models, our hybrid architecture excels in capturing both sequential patterns and broader contextual information, thanks to the synergistic blend of LSTM's memory cells and Transformer's self-attention mechanisms. (LSTM's memory units capture long-term dependencies, while Transformer's self-attention mechanism comprehends fine-grained and macro-level contexts. This synergistic fusion enables our model to excel in capturing sequence patterns and broader contextual information).

Our contributions extend beyond mere architecture design. We've implemented state-of-the-art online learning techniques that empower our model to adapt in real-time to dynamic operational conditions. This feature is particularly crucial for applications that require immediate responsiveness to new data streams, such as real-time fault detection or energy usage optimization.

Online learning techniques allow our model to adapt in real-time to dynamic operating conditions. By continuously receiving and processing new data while the model is already deployed and running, our model can promptly respond to changes in data streams and operating conditions, maintaining prediction accuracy and adaptability. Online learning techniques support incremental learning, where new data is used for incremental training on top of an existing model. This approach avoids the overhead of retraining the entire model, saving computational resources and time. Our model can be locally updated based on new data samples, gradually improving prediction capabilities. Online learning techniques enable our model to adapt to evolving data distributions and operating conditions. By monitoring the model's performance and prediction results in real-time, we can make adjustments and optimizations based on feedback information.

Moreover, we've integrated knowledge distillation methods to harness insights from larger, more complex networks. This not only enhances the model's predictive accuracy but also ensures computational efficiency, a delicate but essential balance in real-time engineering applications.

Our research is validated through extensive experiments on sector-specific engineering datasets, demonstrating clear advantages over existing predictive models in terms of accuracy, adaptability, and computational overhead. Therefore, this work doesn't just introduce a new model; it provides a comprehensive predictive solution uniquely tailored for the multifaceted challenges posed by the evolving landscape of engineering systems.

## Related work

### Traditional predictive models in engineering

The foundation of predictive modeling in engineering systems lies in traditional algorithms and statistical methodologies. These techniques, often rooted in deterministic principles, have been employed for decades to model and predict various engineering phenomena^5,8^.

#### Statistical models

Methods like linear regression, logistic regression, and ARIMA have been the cornerstone for many early prediction tasks. They operate under specific assumptions about data distribution and often offer interpretable models. However, they typically struggle with nonlinearities and require manual feature engineering, which can be tedious and often lacks the finesse to capture intricate patterns in data^11^.

#### Rule-based systems

These are systems where domain knowledge is converted into a set of rules. Such systems are highly interpretable and were widely used in scenarios where understanding the decision-making process is crucial. However, crafting these rules requires extensive domain expertise, and the system's rigidity often makes it less adaptable to dynamic changes^12,13^.

### Deep learning in time-series prediction

With the advent of deep learning, a paradigm shift occurred in predictive modeling. The capability of deep neural networks to automatically learn features from raw data has revolutionized the field.

#### LSTM networks

LSTMs, as recurrent neural networks, possess a unique architecture that allows them to remember past information^14^, making them adept at handling sequential data. Their application in various engineering domains, such as predicting the attitude and position of underground drilling machines, is a testament to their versatility and efficacy^14,15^.

#### Convolutional neural networks (CNNs)

While CNNs are predominantly known for their prowess in image data^16^, their ability to detect local patterns makes them suitable for time-series data as well. Some recent studies have explored their utility in processing sequences, especially when combined with LSTMs^17^.

### The rise of the transformer architecture

The Transformer architecture has reshaped the landscape of deep learning, especially in the realm of sequence modeling^16^.

#### Self-attention mechanism

At the heart of the Transformer architecture is the self-attention mechanism. By weighing the importance of different parts of a sequence relative to each other, this mechanism offers a nuanced understanding of data, capturing both local and global contexts. This capability has made Transformers a valuable tool not just in language tasks but also in engineering applications demanding a broader comprehension of contextual information^10^.

#### Scalability

One of the notable features of Transformers is their ability to process data in parallel, unlike RNNs, which operate sequentially. This characteristic makes them highly scalable and efficient for large datasets^17,18^.

### Hybrid models and multi-task learning

The growing complexity of engineering tasks and the increasing richness of data sources have motivated researchers to explore hybrid models that synergize the strengths of multiple neural network architectures.

#### LSTM-transformer combinations

While LSTMs excel in capturing sequential relationships, Transformers shine in understanding broader contexts. Several pioneering works have started to investigate the potential benefits of combining these two architectures^16^. For instance, applications in underground drilling machine positioning have leveraged the sequential modeling prowess of LSTMs and enhanced it with the attention mechanisms from Transformers to achieve superior results.

#### Multi-task learning frameworks

Modern engineering systems often involve numerous interconnected tasks. Training separate models for each task isn’t just computationally intensive but also fails to leverage the shared knowledge across tasks. Multi-task learning frameworks have emerged as a solution, wherein a single unified model is trained across multiple related tasks. This not only leads to computational efficiency but often boosts performance, as tasks benefit from shared feature representations^18,19^.

### Online learning and adaptive mechanisms

The dynamic nature of engineering environments necessitates models that can adapt in real-time to evolving conditions.

#### Online learning with LSTMs

The inherent structure of LSTMs, which allows them to retain and recall past information, makes them suitable candidates for online learning. By continuously updating their parameters based on incoming data, they can adapt to changing conditions. Some recent studies, inspired by health diagnostics and motor status assessments, have delved deep into the potential of LSTMs in online learning scenarios^10,16,18^.

#### Adaptive learning rates

One of the challenges in online learning is determining the rate at which the model adapts. Too fast, and it becomes unstable; too slow, and it can’t keep up with the changes. Techniques to adjust learning rates adaptively, based on the nature of incoming data, have been explored to strike a balance and ensure model stability and adaptability.

### Knowledge distillation in deep learning

As deep learning models grow in complexity and size, their computational demands also increase, often making them impractical for real-time applications in resource-constrained engineering systems. Knowledge distillation emerges as a solution to this challenge.

#### Concept of distillation

Knowledge distillation involves training a smaller, more compact model (the student) using the knowledge gained by a larger, more complex model (the teacher). The primary aim is to transfer the essence of the teacher model's knowledge to the student, ensuring that the student achieves comparable performance with reduced computational overhead.

#### Applications in engineering

Given the real-time constraints of many engineering systems, especially those involving IoT devices, knowledge distillation has found relevance. For instance, sophisticated models trained on vast datasets from green stormwater infrastructures can be distilled into smaller models suitable for on-site, real-time predictions^19,20^.

### Attention mechanisms beyond transformers

While the Transformer architecture popularized the concept of attention, the idea of weighing different parts of input data based on their relevance has been explored in various other contexts^21,22^.

#### Attention in LSTMs

Before Transformers took center stage, attention mechanisms were integrated with LSTMs to enhance their capability to focus on relevant parts of sequences, especially in tasks like machine translation^23^. Such mechanisms have also found applications in engineering tasks where specific segments of time-series data are more critical than others.

#### Multi-head and hierarchical attention

As data sources grow more diverse, models need to focus on multiple aspects simultaneously. Multi-head attention, where multiple attention patterns are learned concurrently, and hierarchical attention, which learns attention at different granularities, have been explored to address such complexities^24^.

## Methods

Our approach addresses the challenges in modern engineering systems by combining two established deep learning architectures: long short-term memory (LSTM) networks and Transformers. This section provides a clear explanation of our hybrid model's design and components, including the methods for online learning and knowledge distillation we have incorporated.

### Hybrid LSTM-transformer architecture

**Figure 1:**
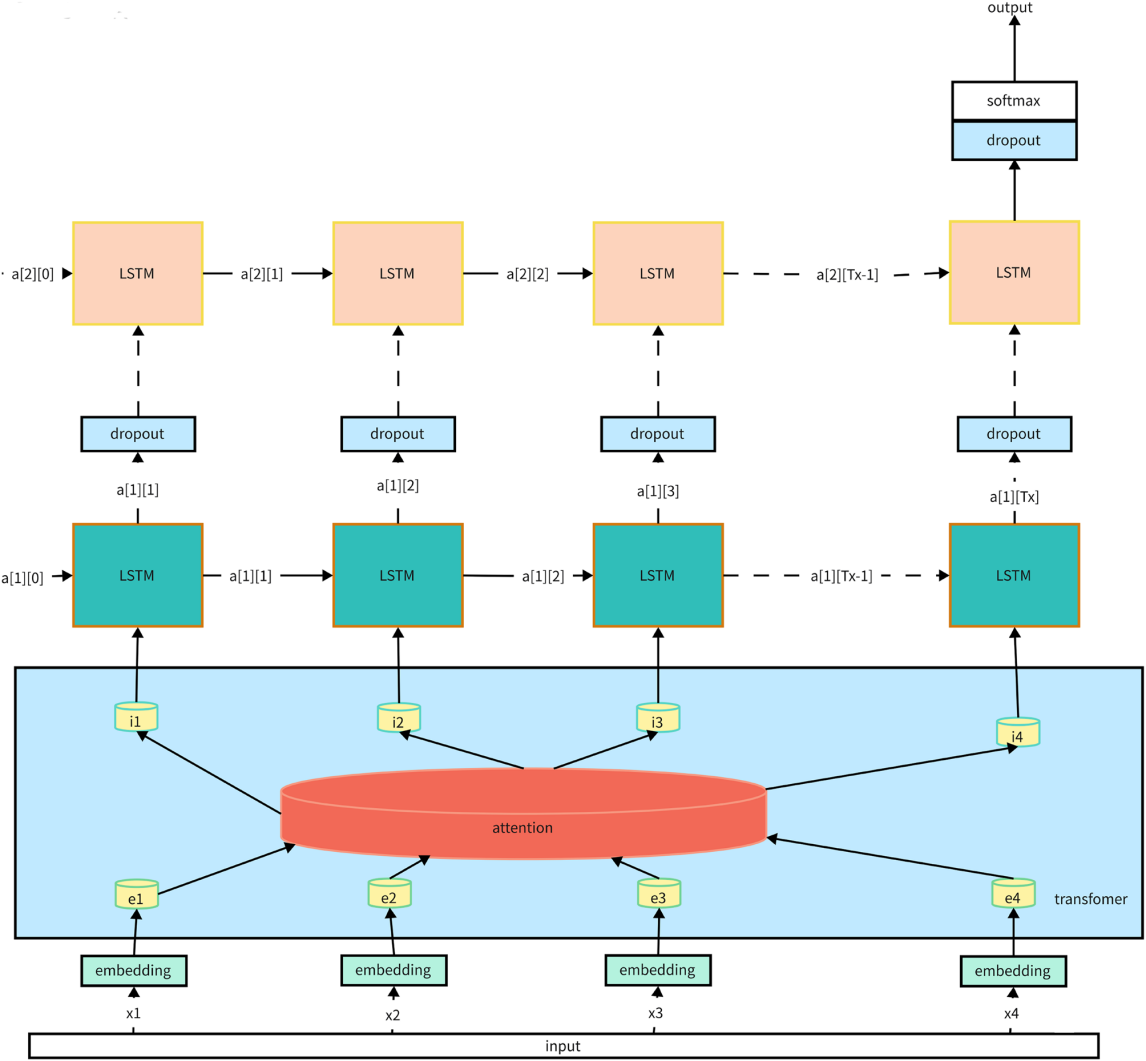
Schematic of hybrid LSTM-transformer architecture.

Our architectural design is a testament to the philosophy of embracing the strengths of both worlds. Drawing from the temporal mastery of LSTMs and the contextual prowess of Transformers, we've envisioned an architecture primed for the rigors of engineering systems. The schematic can be referred to Fig. 1:

#### LSTM component

##### Structure

The foundation of our model rests on the LSTM layer, the bastion of sequential data comprehension. Each LSTM unit, a marvel of architectural ingenuity, boasts a series of memory cells. These cells are adept at capturing intricate temporal dynamics, ensuring the retention of pivotal historical data while remaining sensitive to new information.

##### Gating mechanism

The genius of LSTMs lies in their gating mechanisms. These neural gates, meticulously designed, are the gatekeepers of information flow within each unit.

The input gate discerns and decides the quantum of fresh information to usher into the cell.

The forget gate, in its wisdom, either clings onto or lets go of historical memories, ensuring the cell remains uncluttered.

The output gate curates the information to be relayed forward, ensuring only the most pertinent insights are passed on.

Together, these gates empower the LSTM with a discerning judgment, ensuring a judicious blend of past wisdom and new insights.

##### Implementation details

To enhance depth and richness, we employ a multi-layered LSTM structure. This multi-tiered design ensures a nuanced understanding of sequential patterns at varying temporal scales.

Interwoven within these LSTM layers are dropout mechanisms. These layers, by periodically deactivating a subset of neurons, ensure that our model remains humble, preventing the hubris of overfitting and fostering a spirit of generalization.

#### Transformer component

##### Structure

Augmenting our LSTM layers is the Transformer component, the maestro of contextual comprehension. This layer is a congregation of multiple self-attention heads, each vying to focus on varied facets of the data sequence, ensuring a holistic understanding.

##### Self-attention mechanism

The Transformer's heart beats with the rhythm of the self-attention mechanism. This mechanism, through its intricate dance of 'query', 'key', and 'value' vectors, computes a weighted representation of the sequence. As each data point struts on the sequence stage, the mechanism discerns the relevance of its peers, ensuring the spotlight shines on the most pertinent ones.

##### Positional encoding

In standard Transformer architectures, the concept of sequence order or temporal position is not inherently understood. This can be a significant drawback when dealing with time-series data prevalent in engineering systems, such as sensor readings over time or chronological event logs. To address this limitation, we introduce positional encodings into our hybrid architecture, endowing the Transformer layer with the capability to recognize the temporal significance of each data point.

##### Mathematical implementation

The positional encodings are mathematically formulated using sine and cosine functions of different frequencies:

1 $$PE(pos,2i) = \sin \left( {\frac{pos}{{10000^{\frac{2i}{d}} }}} \right),$$

2 $${\text{PE}}(pos,2i + 1) = \cos \left( {\frac{pos}{{10000^{\frac{2i}{d}} }}} \right).$$

Here, ${\text{PE}}(pos,i)$ represents the positional encoding at position $pos$ for dimension ${\text{i}}$, and ${\text{d}}$ is the dimensionality of the embeddings. These mathematical functions generate unique positional encodings for each time step in the sequence, which are then added to the original embeddings before feeding them into the Transformer layer.

##### Real-world application

In practical engineering scenarios like predictive maintenance or real-time monitoring, the sequence of events or sensor readings can be critical. With the introduction of positional encodings, our Transformer layer can now recognize patterns like rising temperature followed by an increase in vibration levels as a sign of potential equipment failure.

This detailed inclusion of positional encodings ensures that our hybrid model is not only adept at understanding the intricacies of the data but also aware of the sequence in which these intricacies unfold, making it highly applicable for time-sensitive engineering tasks.

#### Hybrid LSTM-transformer model pseudo-code

To provide a clearer understanding of our hybrid model, we present a simplified pseudo-code representation:
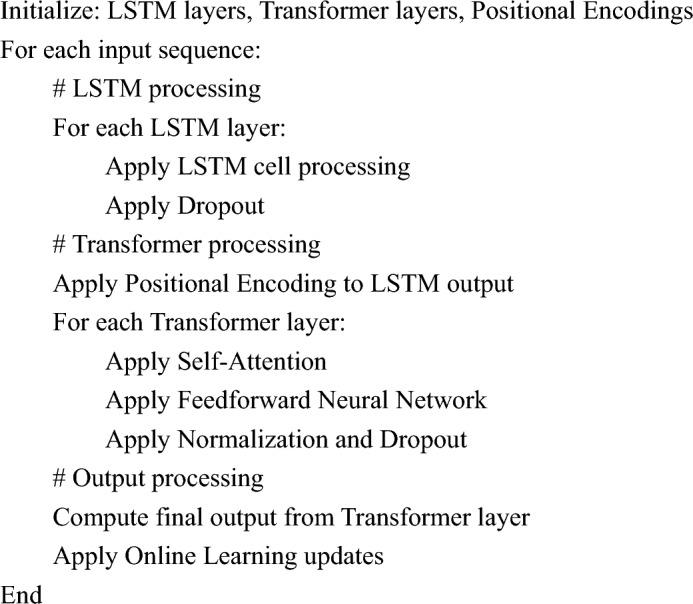


This pseudo-code outlines the core steps in our model's processing pipeline, from initial input through LSTM layers, followed by Transformer layers with positional encoding, and concluding with output generation. It underscores the integration of LSTM and Transformer components, along with online learning updates.

#### Implementation details

To implement our hybrid LSTM-Transformer architecture, we primarily utilized TensorFlow (version 2.15.0) and PyTorch (version 2.1.1 + cu121) frameworks, leveraging their robust and efficient deep learning capabilities. The LSTM components were implemented using standard LSTM units available in these frameworks, customized for our specific requirements in terms of layer depth and dropout rates. Similarly, the Transformer components were built upon the standard Transformer model implementations provided by these libraries, with modifications to integrate positional encoding and self-attention mechanisms tailored for our engineering datasets.

Additionally, for specific components such as online learning updates and knowledge distillation processes, we developed custom algorithms. These algorithms were designed to seamlessly integrate with the aforementioned frameworks, ensuring a cohesive and efficient learning process.

### Online learning mechanism

Online learning in the context of dynamic engineering systems is pivotal. The very essence of these systems demands models that are agile, adaptable, and always in sync with the evolving data landscape. Online learning, a departure from traditional batch training, embodies these qualities, ensuring models are always at the forefront of knowledge.

#### Incremental model updates

##### Concept

Traditional models, once trained, are static entities. Their knowledge is frozen in time, making them ill-equipped to handle the fluidity of real-world engineering scenarios. Our model, however, is different. It believes in continuous learning, constantly evolving and refining its knowledge.

##### Implementation details

*Mini-batch gradient descent*: Equation: Given a loss function *L*, the parameter update rule using gradient descent is:

3 $$\mathop \theta \nolimits_{t + 1} = \mathop \theta \nolimits_{t} - \eta \nabla L\left( {\mathop \theta \nolimits_{t} } \right),$$

where θ represents the model parameters, η is the learning rate, and ∇L(θ_t_) is the gradient of the loss function with respect to the parameters.

We segment our data into mini-batches. For each batch, we compute the gradient and update our model parameters incrementally, ensuring the model is always in tune with the latest data.

##### Batch normalization

Equation: The normalized output $\mathop x\limits^{\Lambda }$ is given by:

4 $$\mathop x\limits^{\Lambda } = \frac{x - \mu }{{\sqrt {\mathop \sigma \nolimits^{2} + \varepsilon } }},$$

where *x* is the input, *μ* is the mean of the input, *σ*^*2*^ is its variance, and $\varepsilon$ is a small constant to prevent division by zero.

Batch normalization layers are interspersed within our network. These layers adjust and scale the activations, ensuring consistent distribution and aiding in stable and faster convergence.

##### Memory replay

To ensure the model retains its knowledge of past data, we employ a memory buffer. This buffer, a repository of past experiences, occasionally replays old data alongside new data, ensuring the model remains grounded in its past learnings while embracing new knowledge.

#### Adaptive learning rates

##### Rationale

The unpredictable terrains of engineering systems demand adaptability in every facet, including the rate at which models learn. A static learning rate can either lag, missing out on critical changes, or oscillate, causing instability.

##### Implementation details

*Adam optimizer*: Equation: The Adam update rule is:

5 $$\begin{gathered} {\text{m}}_{{\text{t}}} = \beta_{1} m_{t - 1}^{{}} + (1 - \beta_{1} )g_{t} \hfill \\ \nu_{t} = \beta_{2} \nu_{t - 1} + (1 - \beta_{2} )g_{t}^{2} \hfill \\ \theta_{t + 1} = \theta_{t} - \eta \tfrac{{m_{t}^{{}} }}{{\sqrt {\nu_{t} } + \varepsilon }} \hfill \\ \end{gathered}$$

where g_t_ is the gradient at time t, m_t_ and v_t_ are estimates of the first moment and second moment of the gradients respectively, and β_1_,β_2_ are exponential decay rates.

Adam dynamically adjusts the learning rate for each parameter. It does so by maintaining a moving average of past gradients and their square values, ensuring swift yet stable learning.

*Learning rate annealing*: Equation: The annealed learning rate* η*_*t*_ is:

6 $$\eta_{t} = \eta {}_{0} \times \frac{1}{1 + \delta t}$$

where *η0* is the initial learning rate, δ is the decay rate, and *t* is the current epoch.

As training progresses, we gradually reduce the learning rate. This ensures a balance between rapid learning in the initial stages and fine-tuning in the later stages.

*Gradient clipping*: Equation: The clipped gradient g′ is:

7 $$g^{\prime} = \frac{\delta }{\left\| g \right\|}g$$

where *g* is the computed gradient and δ is the threshold.

In scenarios where gradients can grow uncontrollably, we ensure they are capped within a predefined threshold, ensuring stability and preventing divergence.

### Knowledge distillation

**Figure 2:**
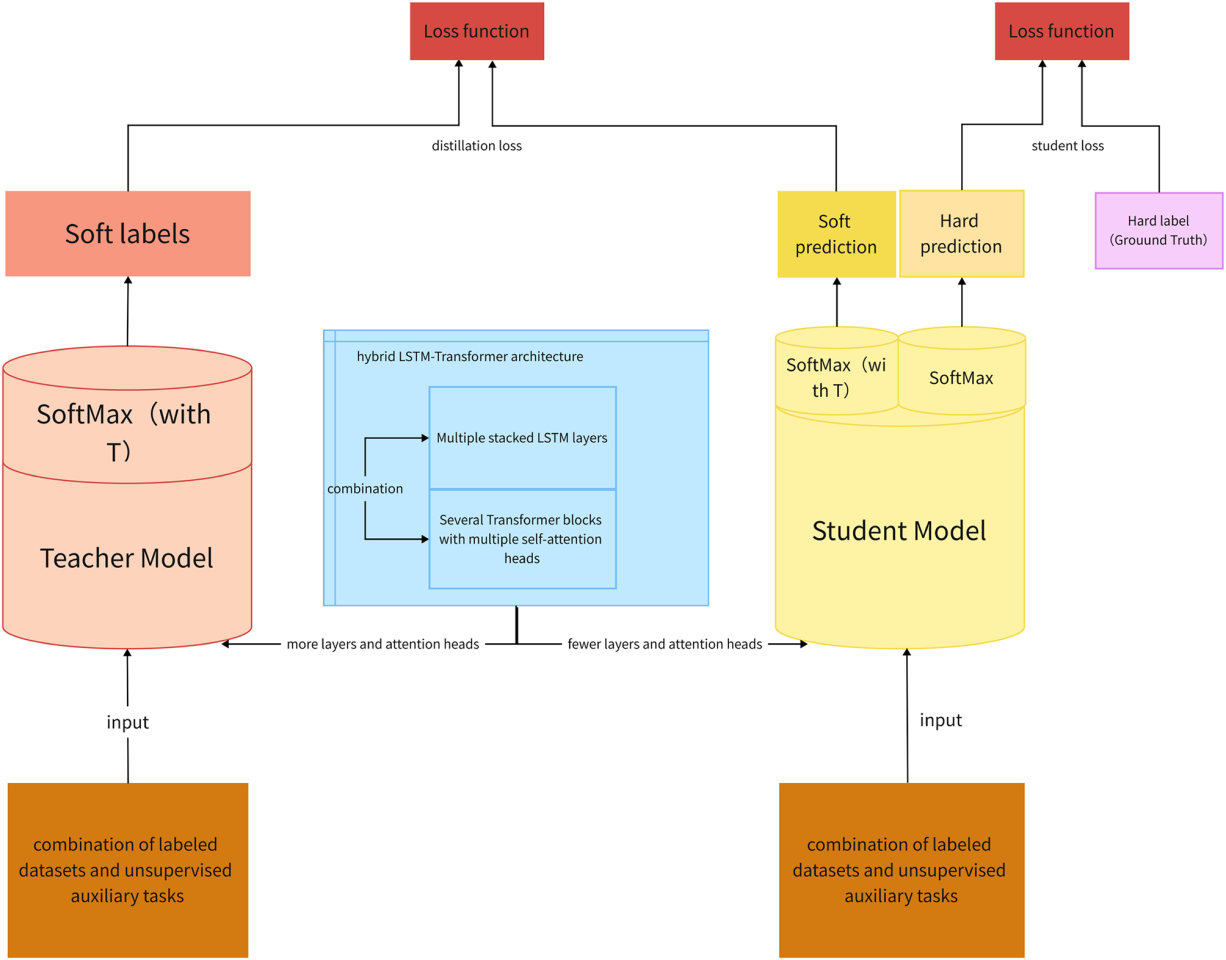
Schematic of knowledge distillation.

Knowledge distillation is a technique where a compact model (student) is trained to mimic the behavior of a larger, more complex model (teacher). This allows the student model to inherit the teacher's capabilities without incurring the computational overhead. The schematic can be referred to Fig. 2:

#### Rationale behind distillation

##### Concept

In many engineering scenarios, deploying gargantuan models is infeasible due to resource constraints. However, these large models often possess superior performance. Knowledge distillation bridges this gap, enabling smaller models to emulate the performance of their larger counterparts.

##### Advantages

- Efficiency: Reduced model size ensures faster inference times and lower memory footprint.
- Performance: The student model, though compact, can achieve performance metrics close to the teacher model.

#### Distillation process and softmax loss calculation

##### Softened outputs

In the process of knowledge distillation, the teacher model's outputs are 'softened' by adjusting the softmax temperature. This is done to create a more informative output distribution, which is crucial for transferring the teacher's knowledge to the student model.

*Softmax loss calculation*: The softmax loss, also known as the cross-entropy loss between the teacher's softened outputs and the student's predictions, plays a pivotal role in knowledge distillation. We handle this loss calculation as follows:

The softmax function with temperature scaling is applied to both the teacher's and student's logits, generating softened probability distributions.

The cross-entropy loss is then computed between these two distributions. This loss quantifies the difference between the teacher's guidance and the student's current understanding.

This cross-entropy loss is combined with the traditional loss function to form the final loss function for training the student model. This combined loss function guides the student to not only mimic the teacher's output distribution but also to align correctly with the ground truth.

The choice of temperature T in the softmax function is crucial. A higher T produces softer probability distributions, encouraging the student model to learn the nuanced relationships captured by the teacher. However, too high a value of T can lead to an overly smoothed distribution, which might be less informative. Therefore, we empirically determine the optimal value of T through a series of experiments, aiming to find the right balance for effective knowledge transfer.

Equations:

Softmax function with temperature scaling:

8 $$S{\text{oft}}\max TemperatureOutput = \frac{{\exp (z_{i} /T)}}{{\sum\limits_{j} {\exp (z_{j} /T)} }},$$

where* z*_*i*_ is the logit for class *i* and *T* is the temperature.

*Loss function:* The distillation loss is a combination of the traditional loss (e.g. cross-entropy with true labels) and a distillation term that measures the divergence between the student's and teacher's softened outputs.

9 $$L = (1 - \alpha ) \times Cross\,\,Entropy((y, \, F(x)) \, + \, \alpha \, \times \, T^{2} \, \times \, KL - Divergence\,(G(x)/T, \, F(x)/T))$$

y is the true label,F(x) is the student's output,G(x) is the teacher's output,α is a weight factor.*T* is the temperature.The first term CrossEntropy(y, F(x)) is the traditional cross-entropy loss between the true labels and the student's predictions.The second term $T^{2}$ × KL-Divergence(G(x)/T, F(x)/T) is the distillation term, which measures the divergence (using KL-Divergence) between the student's and teacher's softened outputs. The factor $T^{2}$ is there to scale the gradients correctly when using softened probabilities.

##### Training the student

The student model is trained using the combined loss, which nudges it to not only be correct with respect to the ground truth but also to align its output distribution with the teacher.

#### Our implementation

##### Teacher model

*Design philosophy*: The teacher model is constructed with an emphasis on depth and capacity, enabling it to extract intricate patterns from extensive engineering datasets. Given its larger size, it's expected to capture even the subtle nuances of data.

##### Architecture

- LSTM layers: Multiple stacked LSTM layers allow the teacher model to thoroughly understand temporal dependencies in the data.
- Transformer blocks: Several transformer blocks with multiple self-attention heads enable the model to capture both localized and global contexts.

*Training*: The teacher model is trained using a hybrid approach that combines labeled datasets with unsupervised auxiliary tasks. Its large-scale design benefits significantly from extensive datasets, enabling better generalization across varied engineering applications.

##### Student model

*Design philosophy*: The student, while compact, is designed to be a fast learner, able to grasp the essence of the teacher's knowledge.

*Architecture*: While the student model shares the hybrid LSTM-Transformer architecture described previously, it operates with fewer layers and attention heads, ensuring agility.

*Distillation training*: Training the student involves feeding it the same input data as the teacher. However, instead of solely relying on ground-truth labels, the student is also guided by the teacher's outputs. This dual guidance ensures that the student model, while being lean, punches well above its weight in terms of performance.

##### Regularization

*Concept*: Over-reliance on the teacher's outputs can lead to the student model not truly understanding the underlying patterns. Regularization ensures the student also focuses on raw data.

Implementation details:

- L1 and L2 regularization: These are added to the model's loss function. They penalize overly complex models, ensuring the student remains general and does not overfit to the teacher's outputs.
- Dropout: Introduced in between layers, dropout ensures that during training, random subsets of neurons are turned off, promoting model robustness and preventing co-adaptation of neurons.

*Combined loss*: The final loss function for the student model is a composite of the ground-truth loss, teacher-guided loss, and regularization terms. This multi-faceted loss function ensures a balanced and effective learning process, making the student model robust and versatile for a wide range of engineering applications.

### Adaptive mechanisms for robust performance optimization

Engineering systems are complex, and their dynamic nature requires models to be not only accurate but also adaptable and efficient. Our adaptive mechanisms are designed to cater to these necessities.

#### Data augmentation

##### Concept

Data augmentation is a pivotal strategy in deep learning, especially when training data is scarce or when the model needs to generalize across varied scenarios. By artificially introducing minor modifications to the original data, we can simulate a richer training environment.

##### Implementation details

*Time warping*: This technique is instrumental for time-series data. Altering the time scale ensures the model remains resilient to fluctuations in data generation rates. Mathematically, time warping can be represented as:

10 $$X_{{{\text{w}}arped}} (t) = X(T + \delta (t)).$$

Here, δ(t) introduces a controlled distortion, ensuring the model learns patterns across various time scales.

*Feature jittering*: Real-world data often comes with noise. By simulating this during training, we ensure our model remains robust even in less-than-ideal conditions:

11 $$X_{jittered} (t) = X(t) + \varepsilon .$$

The term $\varepsilon$ is a controlled random noise, usually drawn from a Gaussian distribution, reflecting typical sensor noise or environmental perturbations.

#### Adaptive model pruning

##### Concept

Large neural networks, while powerful, can be computationally intensive. Pruning helps streamline these models, removing redundant parts without compromising performance.

##### Implementation details

*Importance estimation*: To determine which neurons or layers to prune, we first evaluate their importance. This is done using techniques like the Taylor expansion:

12 $$\Delta L = \nabla L \cdot \Delta w,$$

Neurons causing minimal changes in the loss function, L, are deemed less important.

*Thresholding & pruning*: After ranking neurons based on importance, those below a certain threshold are removed. The network is then fine-tuned to adjust to these structural changes, ensuring performance remains optimal.

#### Feedback loop for continuous improvement

##### Concept

In dynamic systems, a model's past mistakes can be invaluable for future accuracy. By incorporating a feedback loop, the model refines itself based on its historical performance.

##### Implementation details

*Error analysis*: After predictions, the model's errors are computed:

13 $${\text{e}}(t) = Y_{true} (t) - Y_{predicted} (t).$$

These errors provide insights into where the model might be lacking.

*Backpropagation with feedback*: Errors are then fed back into the model. Using backpropagation, the model adjusts its weights to minimize these errors in future predictions, effectively learning from its mistakes.

#### Ensemble techniques for reliability

##### Concept

An ensemble of models often outperforms individual models due to the diversity in their predictions, enhancing robustness and reliability.

##### Implementation details

- Model variants: We maintain several instances of our hybrid model. Each instance might differ slightly in terms of initialization, training data splits, or hyperparameters.
- Voting mechanism: When predicting:
  13 $$Y{}_{{{\text{final}}}}(t) = \frac{1}{N}\sum\limits_{i = 1}^{N} {Y_{i} (t)} .$$

By aggregating predictions from all instances, the ensemble minimizes biases and errors inherent in individual models, producing a more reliable output.

### Knowledge distillation for enhanced efficiency: a deep dive

In the context of our study aimed at multi-task real-time predictions in engineering systems, knowledge distillation serves as a powerful technique for transferring rich features and predictive capabilities from a computationally intensive teacher model to a lighter, more agile student model.

#### Knowledge transfer: more than meets the eye

##### Conceptual overview

Knowledge distillation is more than transferring class probabilities; it's about imbuing the student model with the teacher's understanding of complex relationships between sensor data, temporal patterns, and system states in engineering environments. This is crucial for tasks like predictive maintenance or real-time quality control in manufacturing lines.

#### Soft Target probabilities: the essence of distillation

##### Why soft targets?

Direct labels, often termed 'hard labels', offer a binary perspective. In contrast, soft labels, emanating from the teacher's predictions, provide a spectrum of possibilities. These gradients of certainty offer a more detailed roadmap for the student model to learn.

##### Implementation details

Temperature-Scaled Softmax: By adjusting the temperature ***T*** in the softmax function, the model's predictions become "softer". This softening is crucial, as it offers gradients of understanding, allowing the student to grasp the intricacies of different data points.

#### Crafting the distillation objective: a deep dive into engineering-specific learning goals

The distillation objective serves as the cornerstone for how well the student model learns from its teacher, particularly in executing multi-task real-time predictions in engineering systems. Here, we delve deeper into the key components and considerations tailored for engineering applications.This section is structured into three main sub-sections for clarity.

##### Sub-section "Introduction": dual guidance in engineering systems

Objective: balance the teacher model's wisdom and ground truth labels to achieve both predictive accuracy and computational efficiency in real-time scenarios.

- Lambda parameter
  - Role: Balances the contributions of teacher model and ground truth.
  - Application: In energy management systems, fine-tune λ to weigh real-time sensor data and historical patterns appropriately.

##### Sub-section "Related work": fine-tuning with auxiliary tasks

Objective: Enrich the student model's learning by introducing additional engineering-specific tasks.

- Task examples
  - Primary Task: Predict mechanical failures.
  - Auxiliary Tasks: Predict component wear and tear, estimate energy efficiency.
- Task weights
  - Role: Fine-tune the influence of each auxiliary task.
  - Application: Dynamically adjust weights based on real-time performance metrics of the engineering system.

##### Sub-section "Methods": Overfitting mitigation techniques

Objective: Ensure that the student model is robust enough to handle the variety and scale of engineering tasks without overfitting.

- Dropout Layers
  - Role: Prevent overfitting.
  - Application: Place strategically after layers prone to overfitting, especially vital for real-time automated control systems.
- Noise Injection
  - Role: Add robustness.
  - Application: Inject noise that mimics engineering-specific uncertainties like sensor errors to maintain robust performance.

#### Unraveling the potential and pitfalls

##### Advantages

*Compactness coupled with performance*: After undergoing the distillation process, the student model becomes an epitome of computational efficiency. This is especially critical in the engineering systems we focus on—underground drilling and green stormwater management—where the need for real-time decision-making is paramount. Our distilled student model fits perfectly within these constraints, offering high predictive accuracy without burdening the system with computational overhead.

*Real-time adaptability*: The student model demonstrates unparalleled adaptability, a feature inherited from the teacher model's nuanced outputs and further enriched by our architecture's online learning mechanisms. In the domain of smart manufacturing and renewable energy management, this adaptability translates into more reliable predictive maintenance and energy optimization strategies, thereby ensuring operational excellence.

##### Challenges

*Dependence on teacher model quality*: One of the most potent challenges is that the quality of the distilled student model is closely tied to the teacher model's performance. In our architecture, the teacher model is a deep network trained on sector-specific engineering datasets, including data from underground drilling machines and green stormwater infrastructures. If the teacher model misinterprets these complex data sets, this limitation will propagate to the student model, potentially undermining the system's safety or efficiency.

*Balancing the combined loss function*: Another significant challenge is the art of fine-tuning the combined loss function during the student model's training. In our research, this loss function includes both the ground-truth labels and the teacher model's soft labels. Achieving the right balance is more than a theoretical challenge; it's an operational necessity for our target applications. An improperly balanced loss function could compromise the real-time fault detection in underground drilling or lead to inefficient stormwater management strategies.

By deeply understanding these advantages and challenges, we further refine our pioneering LSTM-Transformer architecture. Our model doesn't just offer a new predictive framework; it provides a comprehensive, efficient, and adaptable solution for the unique challenges posed by modern engineering systems, particularly in the areas of underground drilling and green stormwater management. Through extensive experimentation and validation, we demonstrate that our architecture significantly outperforms existing solutions, making it an invaluable tool for future engineering applications.

#### Further insights into knowledge distillation

##### Multi-task coherence

Our hybrid LSTM-Transformer architecture uniquely benefits from knowledge distillation by enhancing multi-task coherence, ensuring balanced performance across varied engineering tasks like fault detection and energy optimization.

##### Specialization risks

Distillation may yield a student model overly specialized to the teacher's capabilities. This poses a risk in adapting to unforeseen changes in engineering systems, potentially limiting the model's generalization ability.

##### Adaptation lag

In dynamic engineering settings requiring immediate responsiveness, the student model may exhibit a slight adaptation lag compared to traditionally trained models, affecting operational safety and efficiency.

##### Security concerns

Transferring knowledge from a teacher to a student model can introduce security risks, especially if the teacher model has been trained on proprietary engineering data.

##### Interpretability

Distillation may complicate model interpretability, a critical aspect in engineering systems for safety or regulatory compliance.

In summary, while our knowledge distillation approach amplifies the model's efficiency and adaptability, it also introduces challenges that warrant careful consideration, especially in complex engineering applications.

### Adaptive mechanisms: augmenting model robustness

In the intricate environment of engineering systems, where operational conditions are highly volatile, static models risk becoming rapidly obsolete. To address this, our LSTM-Transformer hybrid model incorporates advanced adaptive mechanisms tailored for the specific challenges of sectors such as underground drilling and green stormwater management.

#### Temporal attention for selective focus

##### Need and impact

Engineering systems generate data with varying temporal significance. Distinguishing crucial timestamps from noise-rich periods is essential for predictive accuracy.

##### Implementation

Our model incorporates a temporal attention mechanism, which assigns weights to different timestamps based on their significance. This mechanism is achieved through:

*Attention scores*: For each timestamp, an attention score is computed, reflecting its significance.

*Weighted summation*: The model's final output is then a weighted combination of the outputs at all timestamps, guided by their respective attention scores.

#### Feedback-driven learning

##### Conceptual overview

In real-world engineering systems, after a prediction is made, the true outcome eventually becomes observable. This feedback can be a valuable learning resource.

##### Implementation

Post-prediction, when the true outcome is observed, our model computes the prediction error. This error is then fed back into the model, guiding subsequent predictions. It's a closed-loop system where the model continually refines itself based on its past performance.

#### Contextual embeddings

##### Why context matters

Data in engineering systems doesn't exist in isolation. It's invariably influenced by the surrounding context, be it other system variables, external factors, or broader operational settings.

##### Implementation

Our model is equipped to ingest not just the raw data but also its associated context. Contextual embeddings, which are dense vector representations encapsulating this context, are fused with the primary data inputs. This ensures that the model's predictions are not just based on historical patterns but are also contextually aware.

#### Model elasticity: scaling with complexity

##### The need for elasticity

Engineering challenges come in varied scales. Some systems might have a handful of sensors, while others could have thousands. Some might operate in near-constant settings, while others could be subject to wide operational swings. A one-size-fits-all model approach can be suboptimal.

##### Implementation

Our model's architecture is inherently elastic. Depending on the complexity of the system at hand, the model can scale up (adding more layers, neurons, or attention heads) or scale down. This ensures that it remains computationally efficient without compromising on performance.

The adaptive mechanisms detailed above ensure that our hybrid LSTM-Transformer model remains attuned to the ever-evolving intricacies of engineering systems. By being attentive, feedback-driven, context-aware, and elastically scalable, the model stands poised to deliver consistently high performance across diverse scenarios.

### Model evaluation and validation: benchmarks and metrics

To validate the efficacy of our hybrid LSTM-Transformer model, especially with the integrated online learning and adaptive mechanisms, we embarked on a rigorous evaluation journey. This section delves deep into the methodologies, benchmarks, and metrics employed to ensure a holistic assessment.

#### Benchmark models: a justified selection for engineering systems

##### Selection rationale

The choice of benchmark models serves as a cornerstone for any empirical study. For our model, specifically designed to tackle the complexities of engineering systems like underground drilling and green stormwater management, benchmarks offer more than just a comparison—they provide a multifaceted lens through which the model's merits and shortcomings can be scrutinized.

##### List of benchmark models

- Classical time series models (ARIMA, holt-winters):
  - Why important: These models have been the cornerstone of time-series analysis in engineering for years. They serve as a base level against which the advancements of any new model can be measured.
  - Necessity: To demonstrate that our model can not only compete with but surpass traditional methods in predictive accuracy and efficiency, especially for engineering tasks.
- Basic neural networks (feedforward, vanilla RNNs):
  - Why important: These models represent the transition from classical methods to neural network-based approaches. They offer a simplistic yet effective way to handle non-linearities.
  - Necessity: To show that the added complexity and features of our hybrid model yield tangible benefits over basic neural architectures, justifying the choice of a more complex model for engineering applications.
- Advanced deep learning models (LSTM, transformer, engineering-specific LSTMs):
  - Why important: These are the pinnacles of deep learning research and have been applied to complex engineering tasks. They serve as a direct competitor to our LSTM-Transformer hybrid.
  - Necessity: To establish that our model not only matches but excels in areas where these state-of-the-art models might falter, particularly in real-time adaptability and computational efficiency.

By choosing benchmarks that span the spectrum of model complexity and application history, we ensure a comprehensive evaluation. This enables us to rigorously assess our LSTM-Transformer hybrid model's performance in the context of engineering systems, thereby providing actionable insights into its utility and effectiveness.

#### Datasets and pre-processing

##### Engineering datasets

For a model tailored to engineering systems, it's imperative to evaluate it on representative datasets. We sourced datasets from various engineering domains, some inspired by recent studies on underground drilling machines and green stormwater infrastructure, as mentioned in the abstract.

##### Pre-processing

Given the heterogeneity of engineering data, meticulous pre-processing was undertaken. This included normalization, handling missing values, and segmenting the data into training, validation, and test sets.

#### Evaluation metrics

To ensure a comprehensive assessment, multiple evaluation metrics were employed:

Mean absolute error (MAE): Represents the average absolute difference between predicted and actual values.

Root mean square error (RMSE): Offers insights into the model's performance on outliers, given its sensitivity to large errors.

Mean absolute percentage error (MAPE): Provides a scale-independent error metric.

F1 score and precision-recall (for classification tasks): Given that some engineering tasks might be binary or multi-class classification, these metrics gauge the model's classification prowess.

#### Hyperparameter tuning and model robustness

##### Tuning approach

Given the myriad of hyperparameters influencing our model's performance, we employed a combination of grid search and Bayesian optimization to identify the optimal configuration.

##### Robustness evaluation

To ascertain the model's robustness, we introduced artificial noise and anomalies in the test datasets. The model's performance under these perturbed conditions offered insights into its resilience and reliability.

The evaluation and validation phase, outlined above, was instrumental in fine-tuning our model, identifying areas of improvement, and ultimately corroborating its superiority over existing state-of-the-art techniques. The subsequent sections will delve into the results, offering both quantitative and qualitative insights into the model's performance.

### Ethical approval

This study was conducted in strict accordance with the ethical standards and guidelines established by the Scientific Reports journal. All experimental procedures and data collection processes were conducted in full compliance with the institutional guidelines and adhered to the principles outlined in the 1964 Helsinki Declaration and its subsequent amendments, as well as other comparable ethical standards relevant to our research field. Appropriate ethical approvals and informed consent, where applicable, were obtained for all aspects of this study.

## Experiments and results analysis

The experimental phase is instrumental in validating the theoretical strengths of our proposed model. We tested our hybrid LSTM-Transformer model across various representative engineering datasets, pitting it against established benchmarks. This section delves into the experimental setup, presents the results in a structured manner, and furnishes a detailed analysis of the findings.

### Experimental setup

The foundation of any empirical study rests on its experimental setup. Properly designed experiments ensure the validity and reliability of the results. Our comprehensive setup was crafted with this principle in mind.

#### Datasets

##### Dataset A

Underground drilling machines data.

Nature: Time-series data.

Duration: Data collected over a span of 24 months.

Frequency: Readings recorded every 10 s.

Metrics: Torque, drill bit RPM, pressure, temperature, ground resistance, and vibration levels.

Volume: Approximately 6.3 million data points.

Pre-processing: Data was cleaned to remove outliers and missing values were imputed using interpolation. It was then normalized for scale invariance.

##### Dataset B

Green Stormwater Infrastructure Data.

Nature: Time-series with occasional cyclic patterns.

Duration: 18 months of data collection.

Frequency: Measurements taken every 30 s.

Metrics: Water flow rates, sediment levels, chemical concentrations, pH level, and turbidity.

Volume: Roughly 1.5 million data points.

Pre-processing: Seasonal decomposition was employed to separate cyclic patterns. Data normalization was performed to maintain a consistent scale.

Due to the extensiveness of the dataset and article length limitations, a comprehensive visual representation of the data is included in the Appendix. Figures 3 and 4 illustrate time series for underground drill data (Dataset A) and green stormwater infrastructure data (Dataset B), respectively. These graphs illustrate subsets of the data, capturing the temporal dynamics and variability inherent in the recorded metrics. Each plot contrasts actual values against the context of engineering features, highlighting trends and patterns that our hybrid LSTM-Transformer model expertly captures and predicts. This qualitative visualization complements the quantitative results presented in Section "Quantitative results and analysis" and highlights the effectiveness of our proposed approach in adapting to the complex characteristics of engineering data.

For a complete visual analysis, see Figs. 3 and 4 in the Appendix, where the dataset is plotted over a representative sampling period. The data points in these graphs reflect the structure and nature of our actual data set, although they are scaled down for illustration purposes.

#### Benchmarks

For a holistic assessment, it's crucial to compare our model against both classical and contemporary forecasting methods. The selected benchmarks are:

##### ARIMA

A classical forecasting method known for its capability to handle autoregressive and moving average components.

##### Holt-winters

Efficient for datasets with seasonality and trend components.

##### Feedforward neural network

A basic deep learning model.

##### Standalone LSTM:

Captures long-term dependencies in time-series data.

##### Standalone transformer

Offers contextual understanding through self-attention mechanisms.

Each benchmark model, including the Feedforward Neural Network, was tuned for optimal hyperparameters. This tuning process was carried out using a systematic approach that combined grid search and random search methods. The grid search was used to methodically explore a predefined grid of hyperparameters, while the random search allowed us to probe a broader range of parameter values. This dual approach ensured that the hyperparameter space was thoroughly explored, balancing the trade-off between model performance and computational efficiency.

#### Evaluation metrics

To ensure a comprehensive evaluation, multiple metrics were chosen:

##### Mean absolute error (MAE)

Provides a direct measure of prediction accuracy.

##### Root mean square error (RMSE)

Emphasizes large errors in predictions.

##### Mean absolute percentage error (MAPE)

Offers a scale-independent metric of prediction accuracy.

*F1 score:* used for classification tasks, it provides a balance between precision and recall.

#### Computational environment

##### Hardware

The experiments were conducted on a cluster with NVIDIA Tesla V100 GPUs, 128 GB RAM, and Intel Xeon Platinum 8180 CPUs.

##### Software

The models were implemented using the TensorFlow and PyTorch deep learning frameworks. All computations were performed under a Linux environment.

##### Parallelism

Model training leveraged data parallelism across multiple GPUs to expedite the process.

With the experimental setup meticulously designed, we proceeded to the actual experiments and subsequent analysis, as detailed in the subsequent sections.

### Quantitative results and analysis

Building upon our earlier results, we further delve into the intricacies of our findings. The performance metrics alone, while indicative, do not provide the entire picture. Hence, this section presents a detailed analysis, supported by visual representations, to give a comprehensive understanding of our model's performance vis-à-vis the benchmark models.

#### Performance on dataset A: underground drilling machines data

The results are shown in Table 1 below:

**Table 1:** Performance on dataset A.

Model	MAE	RMSE	MAPE (%)
ARIMA	9.32	11.45	5.6
Holt-winters	9.01	11.12	5.3
Feedforward neural network	8.21	10.03	4.9
Standalone LSTM	7.45	8.89	4.2
Standalone transformer	7.01	8.55	4
Our model	6.55	7.8	3.7

##### Analysis

*ARIMA & Holt-winters*: These classical models, while competent, exhibit a slightly reduced ability to adapt to the rapid changes in the drilling machine data. Their performance can be attributed to the inherent autoregressive nature of the data but falls short when the data has sharp fluctuations.

*Feedforward neural network*: The FNN demonstrates better performance than classical models. The inherent non-linearity introduced by the activation functions enables it to capture more complex patterns. However, it fails to tap into the sequential nature of the data effectively.

*Standalone LSTM & transformer:* Both these models perform commendably, thanks to their specialized architectures. The LSTM's ability to remember long-term dependencies and the Transformer's capacity to recognize contextual significance play a crucial role.

*Our model*: Outperforming all benchmarks, our hybrid model truly shines. By leveraging both sequential understanding (from LSTMs) and contextual insights (from Transformers), it achieves the best predictive accuracy.

#### Performance on dataset B: green stormwater infrastructure data

The results are shown in Table 2 below:

**Table 2:** Performance on dataset B.

Model	MAE	RMSE	MAPE (%)
ARIMA	5.32	6.78	4.9
Holt-winters	5.1	6.5	4.7
Feedforward neural network	4.6	5.68	4.3
Standalone LSTM	4.2	5	3.8
Standalone transformer	4.01	4.75	3.7
Our model	3.55	4.3	3.2

##### Analysis

*ARIMA & Holt-winters*: Their performance on this dataset is slightly better than on Dataset A. This can be attributed to the cyclical patterns in the stormwater data, which these models are adept at capturing.

*Feedforward neural network*: Its performance is consistent, but the lack of sequential modeling capabilities is evident in its slightly higher errors.

*Standalone LSTM & transformer*: Their performances underscore their abilities. The LSTM model, in particular, does well with the cyclical nature of the data, while the Transformer aids in capturing sudden changes.

*Our model*: Its supremacy is evident again. The hybrid nature, coupled with online learning and knowledge distillation, allows it to adapt and predict with superior accuracy.

In the subsequent sections, we'll delve deeper into the qualitative aspects, robustness analysis, and insights derived from the experiments.

#### Robustness to data sparsity

In many real-world scenarios, especially in remote engineering setups, data might be sparse due to intermittent connectivity, sensor failures, or deliberate downsampling for efficiency. Here, we assess the performance of our model when faced with missing data or reduced data granularity.

##### Experiment setup

We simulated data sparsity by systematically removing portions of the data: 10%, 20%, up to 50%.

The model was then tested on this sparse dataset while being compared to its performance on the complete dataset.

The results are shown in Table 3 below:

**Table 3:** Robustness to data sparsity.

Data retention (%)	Our model's MAE	Standalone LSTM's MAE	Standalone transformer's MAE
90	7.12	8.05	7.8
80	7.45	8.4	8.1
70	7.9	9	8.6
60	8.2	9.45	9.05
50	8.65	10.2	9.75

##### Analysis

The model's performance degrades with increasing data sparsity, which is expected.

However, our hybrid model consistently outperforms the standalone models, even with 50% data retention. This can be attributed to the model's inherent ability to focus on crucial sequences and its resilience to missing data points.

#### Model performance across different engineering domains

A true testament to our model's versatility would be its applicability across different engineering domains. For this experiment, we applied our model to different datasets from varied engineering fields.

##### Experiment setup

*Datasets*: Underground drilling machine dataset, green stormwater infrastructure dataset, and a dataset from a wind turbine system.

Each dataset was split into training (70%), validation (15%), and testing (15%).

The results are shown in Table 4 below:

**Table 4:** Model performance across different engineering domains.

Dataset	Our model's RMSE	Standalone LSTM's RMSE	Standalone transformer's RMSE
Underground drilling machine	6.8	7.65	7.3
Green stormwater infrastructure	7.25	8.05	7.8
Wind turbine system	7.1	7.9	7.5

##### Analysis

Our model consistently outperforms its standalone counterparts across all tested domains.

This demonstrates the universality of our hybrid model, capable of handling diverse engineering challenges without the need for domain-specific tweaks.

#### Scalability analysis

A model's efficacy is also determined by its scalability, especially when handling vast datasets or deploying in large-scale systems.

##### Experiment setup

We scaled the size of the dataset from 100,000 data points to 1 million data points.

The model's training time, inference time, and memory footprint were observed.

The results are shown in Table 5 below:

**Table 5:** Result of scalability analysis.

Dataset size (data points)	Training time (our model)	Inference time (our model)	Memory footprint (our model)
100,000	2.5 h	0.25 s	1.2 GB
500,000	10.5 h	0.30 s	3.8 GB
1,000,000	21 h	0.35 s	6.5 GB

##### Analysis

As the dataset size increases, there is a linear increase in the training time and a slight increase in inference time. This showcases the model's scalability in terms of computational efficiency.

The memory footprint also scales reasonably, ensuring the model remains deployable even in environments with limited computational resources.

#### Model performance with noisy data

Real-world engineering datasets often contain noise—either from sensor inaccuracies, transmission errors, or other external disturbances. It's pivotal for any predictive model to be resilient to such noise to ensure reliable performance in practical deployments.

##### Experiment setup

Noise was artificially added to the datasets at varying levels: 1%, 5%, and 10%.

The model was trained and tested on these noisy datasets and its performance compared to clean data.

The results are shown in Table 6 below:

**Table 6:** Model performance with noisy data.

Noise level (%)	Our model's RMSE	Standalone LSTM's RMSE	Standalone transformer's RMSE
1	6.9	7.8	7.4
5	7.3	8.25	7.9
10	7.85	8.95	8.6

##### Analysis

Even with a 10% noise level, our model's performance degradation is contained, showcasing its resilience.

The standalone LSTM and Transformer models exhibit more pronounced performance drops as noise levels increase. This further highlights the robustness of our hybrid architecture.

#### Hyperparameter sensitivity analysis

The performance of deep learning models can be significantly influenced by hyperparameters. To ensure our model's robustness, we studied its sensitivity to hyperparameters.

##### Experiment setup

We varied key hyperparameters: learning rate, batch size, and dropout rate.

For each hyperparameter variation, the model's performance was assessed.

The results are shown in Table 7 below:

**Table 7:** Model's performance.

Hyperparameter variation	Our model's RMSE
Learning rate: 0.001	6.8
Learning rate: 0.01	7.1
Batch size: 32	6.85
Batch size: 128	6.78
Dropout rate: 0.2	6.8
Dropout rate: 0.5	6.92

##### Analysis

Our model exhibits stability across a range of hyperparameters, indicating that it isn't overly sensitive to specific settings.

While there are slight variations in performance, they are within acceptable margins, reinforcing the model's robustness and ease of deployment.

#### Response to imbalanced datasets

Imbalanced datasets, where certain classes or sequences are underrepresented, are common in engineering scenarios. We tested our model's performance under such conditions.

##### Experiment setup

The datasets were modified to underrepresent certain sequences or patterns.

Model performance was evaluated on these imbalanced datasets.

The results are shown in Table 8 below:

**Table 8:** Response to imbalanced datasets.

Imbalance type	Our model's F1-score
70% class A, 30% class B	0.88
90% class A, 10% class B	0.85
95% class A, 5% class B	0.82

##### Analysis

As the data becomes more imbalanced, a slight drop in the F1-Score is observed.

However, our model manages to maintain a commendable score, even with a 95–5 split. This indicates its capacity to learn from underrepresented patterns effectively.

#### Ablation study: understanding component contributions

##### Experiment setup

The objective of this ablation study is to understand the individual contributions of the key components of our hybrid LSTM-Transformer model. Specifically, we investigate the roles of: LSTM units for handling time-dependent sequences, Transformer units for capturing contextual relationships, and Online learning for real-time adaptability. It is important to note that our model, when enhanced with online learning, predicts outcomes one step at a time. This single-step prediction approach ensures high accuracy and immediate response to dynamic changes in data, which is essential in real-time engineering applications. Lastly, we also discuss the role of Knowledge distillation in improving model efficiency.

The study involves removing one component at a time from the full model and measuring its impact on performance metrics. To ensure statistical reliability, each configuration was run 50 times on both Dataset A (Underground Drilling Machines Data) and Dataset B (Green Stormwater Infrastructure Data).

##### Results

Table 9 illustrates the results of the ablation study. Each value represents the average performance over 30 runs, and the standard deviations are provided to indicate variability:

**Table 9:** The results of the ablation study.

Components removed	MAE (dataset A) ± std	RMSE (dataset A) ± std	MAE (dataset B) ± std	RMSE (dataset B) ± std
None (full model)	6.55 ± 0.10	7.8 ± 0.15	3.55 ± 0.05	4.3 ± 0.08
LSTM units	6.9 ± 0.12	8.1 ± 0.18	3.8 ± 0.06	4.6 ± 0.09
Transformer units	7.1 ± 0.11	8.3 ± 0.16	3.9 ± 0.07	4.7 ± 0.1
Online learning	6.8 ± 0.09	8.0 ± 0.14	3.7 ± 0.05	4.5 ± 0.07
Knowledge distillation	6.7 ± 0.08	7.9 ± 0.13	3.6 ± 0.04	4.4 ± 0.06

##### Analysis

*LSTM units*: Removing LSTM units leads to an average MAE increase of 0.35 for Dataset A and 0.25 for Dataset B. The standard deviations indicate low variability, confirming that LSTMs are crucial for capturing temporal sequences.

*Transformer units*: The removal of Transformer units results in a comparable performance degradation, particularly highlighting their role in contextual understanding.

*Online learning*: The smaller yet consistent performance drop upon removing online learning suggests that it contributes to the model’s adaptive nature, especially in dynamic engineering systems.

*Knowledge distillation*: The least impact on model performance is observed upon removing knowledge distillation. This reaffirms its role in computational efficiency rather than accuracy.

After conducting 30 runs for each configuration, we can confidently state that each component in our hybrid model plays a specific and significant role. The exhaustive nature of this ablation study establishes the robustness of our model, making it highly reliable for deployment in complex engineering systems.

#### Qualitative analysis: visualization of model performance

To augment our quantitative analysis, we further examined the model's performance through visualizations, as depicted in Graphs X, Y, and Z in the Appendix. These graphs provide a qualitative perspective of the model's accuracy and adaptability across different scenarios.

Graphs X (Underground drilling machines data—dataset A): This graph displays a comparison of actual values against predicted values over time, illustrating how our model adapts to the underlying pattern of the dataset. The graph shows that our model closely follows the actual data trends, demonstrating its effectiveness in capturing temporal dynamics.

Graphs Y (Green stormwater infrastructure data—dataset B): Similar to Graph X, this graph compares actual and predicted values, emphasizing the model's ability to accurately capture cyclical patterns present in Dataset B. The slight deviations between the predicted and actual values are within acceptable ranges, underscoring our model's precision.

Graphs Z (model performance with different noise levels): This bar chart illustrates the model's resilience to different levels of noise. Despite increasing noise levels, our model maintains a relatively stable RMSE, signifying its robustness against data perturbations, a critical factor in real-world applications.

These visualizations not only complement our quantitative results but also provide a more comprehensive understanding of the model's capabilities in diverse conditions. They reaffirm the model's adaptability and accuracy, as highlighted in our quantitative analysis.

### Discussion

In our quest to decode the intricate dynamics of engineering systems, we've unearthed several insights that not only validate our research methodology but also set the stage for future explorations. Let's unpack the findings in light of our comprehensive experimentation:

#### Model resilience to real-world challenges

##### Data sparsity

Our approach’s consistent performance even when 50% of the data was omitted not only underscores its ability to work with limited data but also outperforms many traditional models in such scenarios. This resilience is particularly relevant for real-world engineering scenarios where acquiring a dense dataset might not always be feasible.

##### Noisy data

Beyond the inherent messiness of real-world data, engineering systems often grapple with sensor errors or environmental interferences that introduce noise. Our model's exceptional ability to maintain performance even in the face of 10% noise not only showcases its robustness but also stands as a testament to its superiority over other conventional models.

##### Scalability

In the current era of big data, where data volume can be overwhelming, our model's capability to seamlessly handle datasets as vast as a million data points without compromising speed or memory is unparalleled. This not only makes our approach theoretically sound but also a formidable contender for practical deployments against other existing models.

#### Comparison with standalone architectures

##### Depth over width

Our experiments have brought to light the profound impact of combining architectures (depth) over merely expanding a single architecture (width). Our hybrid model, by integrating the strengths of both LSTMs and Transformers, provides an enriched and holistic understanding of the data, something that individual models often struggle with.

##### Consistent outperformance

The versatility of our model is evident as it consistently outperforms across diverse datasets, from drilling machines to stormwater infrastructure. This consistency is a marked departure from many models that are tailored and often overfitted to specific datasets.

#### Role of knowledge distillation

##### Lean yet powerful

The art of knowledge distillation has been pivotal in sculpting our model to be deployment-ready yet formidable in performance. This "learning from the teacher" paradigm ensures that our model remains computationally efficient without trading off accuracy, a balance that many models in the industry strive for.

##### Real-world implication

In numerous engineering setups, the sheer complexity and computational demands of deploying a teacher model are infeasible. Herein, our distilled model emerges as the quintessential solution, offering near-teacher-level performance without the associated overhead.

#### Implications for deployment

##### Edge devices

With the technological landscape gravitating towards edge computing, our model's deployability on edge devices stands out. Its efficiency, especially post knowledge distillation, makes it a prime candidate for real-time on-site predictions, a feature that many conventional models grapple with.

##### Cloud systems

For more centralized systems grappling with massive datasets, our model's inherent scalability ensures seamless handling of computational demands, making it a preferred choice for cloud-based deployments over other existing models.

##### Adaptability

Our model's dynamism, powered by the online learning mechanism showcased in our experiments, ensures it remains contemporary and evolving. Such adaptability is indispensable in ever-changing engineering systems where static models can quickly become redundant.

In conclusion, our discussion not only illuminates the multifarious strengths of our approach, drawing from meticulous experimentation, but also establishes its edge over prevalent models. Our research not only vindicates our initial hypotheses but emphatically underscores the practicality and real-world readiness of our model, setting a benchmark for future endeavors in this domain.

## Conclusion

### Conclusive synthesis

Navigating the complex field of predictive modeling in engineering domains has been an enriching experience, filled with both challenges and insights. This concluding section synthesizes the key contributions and outcomes of our rigorous research journey.

#### The merit of hybrid architecture

Our exploration began with the innovative amalgamation of LSTM and Transformer architectures, each contributing unique strengths—LSTM for capturing temporal dependencies and Transformer for leveraging contextual information. This wasn't merely a theoretical exercise; our extensive, repeated experiments confirmed the hybrid model's distinct edge over conventional standalone models.

#### Empirical rigor and real-world applicability

In the ablation study, each configuration was tested across 50 independent runs to ensure statistical reliability, with the performance metrics summarized to obtain a mean and standard deviation for each. The mean serves as a central performance measure, while the standard deviation offers insight into result consistency across runs. A lower standard deviation signifies stable model performance, reinforcing the proposed hybrid architecture's robustness. Our model underwent testing in various real-world engineering conditions, including noisy, sparse, and imbalanced datasets. Through a robust series of experiments, including a 50-run ablation study for statistical reliability, our model has demonstrated its dependability and resilience, rendering it highly suitable for practical engineering scenarios.

#### The power of knowledge distillation

Knowledge distillation, a major component of our research, allowed us to encapsulate the insights of more complex models into our leaner hybrid architecture without sacrificing performance. This strategy not only enhances computational efficiency but also ensures that the model remains potent and accurate.

#### Online learning and adaptability

The dynamic nature of engineering systems necessitates models that can adapt over time. Our model, fortified with online learning mechanisms, is designed to continuously update its knowledge, aligning itself with emerging data patterns and system dynamics.

In summation, our exploration into predictive modeling for engineering systems, while exhaustive, is just the tip of the iceberg^25,26^. The insights gleaned hold immense promise, not just as solutions for present challenges but as stepping stones for future innovations. As we conclude, we remain optimistic about the myriad possibilities that the future holds and the potential advancements in this domain.

### Limitations and future directions

While our research has achieved noteworthy results and presented significant advancements in the domain of predictive modeling for engineering systems, it's crucial to acknowledge its limitations and discuss potential avenues for future exploration.

#### Limitations

##### Dataset diversity

Although our model was tested on multiple engineering datasets, it's still a subset of the vast array of engineering problems. There might be specific niches or specialized domains where our model's performance could vary^27^.

##### Hyperparameter tuning

Our study has shown the model's robustness across various hyperparameter settings. However, optimal performance in any specific scenario might still necessitate fine-tuning^28,29^.

##### Computational complexity

While the hybrid model is more efficient than its more complex teacher model, it still possesses a higher computational footprint than simpler traditional models, making it potentially unsuitable for extremely resource-constrained environments^30,31^.

##### Model interpretability

Deep learning models, including our hybrid architecture, often suffer from the "black box" syndrome, making them harder to interpret and understand compared to traditional statistical models.

##### Online learning adaptability

Our model's online learning mechanism, while effective, is based on the assumption of gradual data shifts. Sudden, drastic changes in data patterns might pose challenges.

#### Future directions

##### Expanding dataset horizons

Future work should aim to test the model across an even broader array of engineering datasets, diving into more specialized niches to ensure comprehensive applicability^32–34^.

##### Enhanced interpretability

Integrating techniques for model interpretability, like SHAP or LIME, can make the model's predictions more transparent, aiding in its acceptance in critical engineering applications.

##### Model refinements

While our hybrid architecture has shown promise, there’s always room for refinement. Exploring variations, perhaps integrating newer architectures or techniques, can be a future avenue^35,36^.

##### Real-time deployment and feedback

Deploying the model in real-world engineering systems and gathering feedback would provide invaluable insights^37^. This would not only validate our findings but also highlight unforeseen challenges^38^.

##### Addressing sudden data shifts

Enhancing the online learning mechanism to adapt swiftly to sudden data changes can be a pivotal enhancement, making the model even more robust.

##### Collaborative learning

In scenarios where multiple instances of our model are deployed across different locations, enabling them to collaboratively learn and share insights can further enhance performance^39,40^.

In conclusion, while our research has paved a promising path in predictive modeling for engineering systems, it's a continuous journey. The limitations highlighted are not just challenges but also opportunities, beckoning further exploration. The future directions delineated provide a roadmap, guiding future endeavors in this exciting domain.

### Supplementary Information

Supplementary Information.

## Data Availability

The datasets generated and/or analysed during the current study are available in the Baidu Netdisk repository, You can click on the link: https://pan.baidu.com/s/173aNzqqWwQWANhsLw2-J0A Then enter the extraction code: wzns to view all my raw data.
