# Supplementary material for: Advanced hybrid LSTM-transformer architecture for real-time multi-task prediction in engineering systems
Source: Sci Rep. 2024 Feb 28;14:4890. doi: 10.1038/s41598-024-55483-x (PMC11322354; doi:10.1038/s41598-024-55483-x)
Supplement: Supplementary file 1 — Supplementary Information. [file 41598_2024_55483_MOESM1_ESM.zip › appendix.docx]

**appendix:**


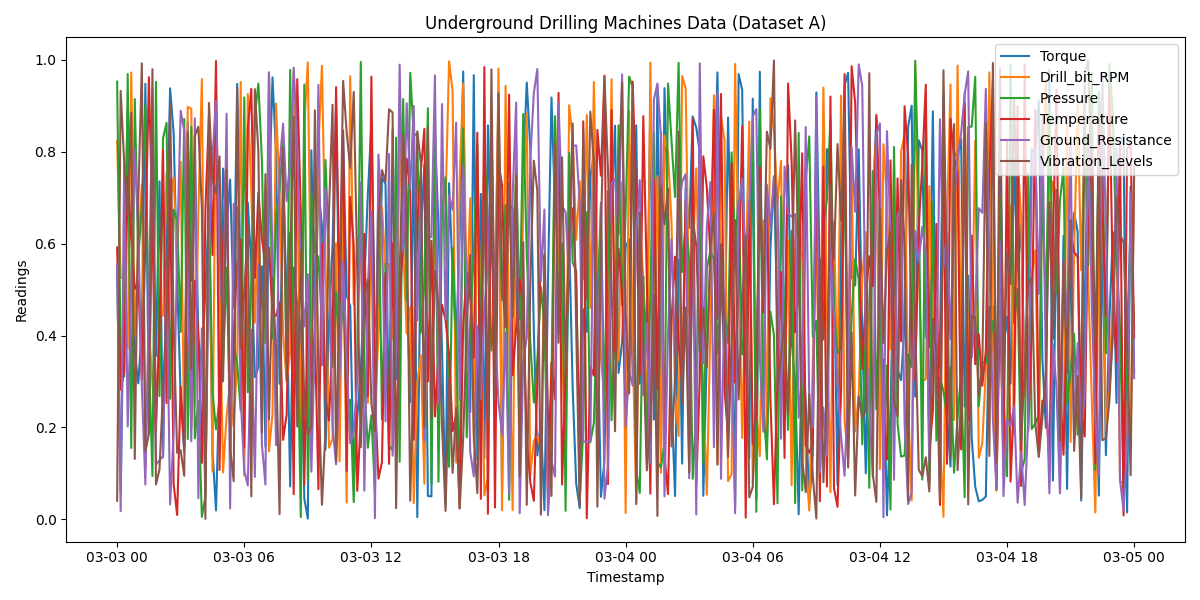


Figure 3.Underground Drilling Machines Data (Dataset A)


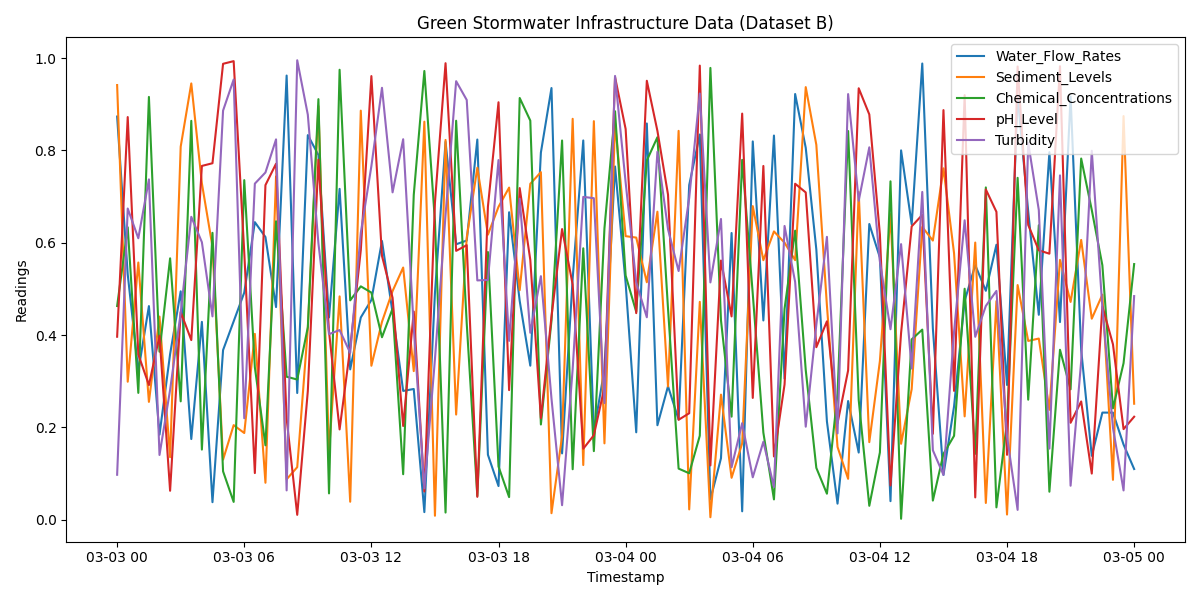


Figure 4.Green Stormwater Infrastructure Data (Dataset B)


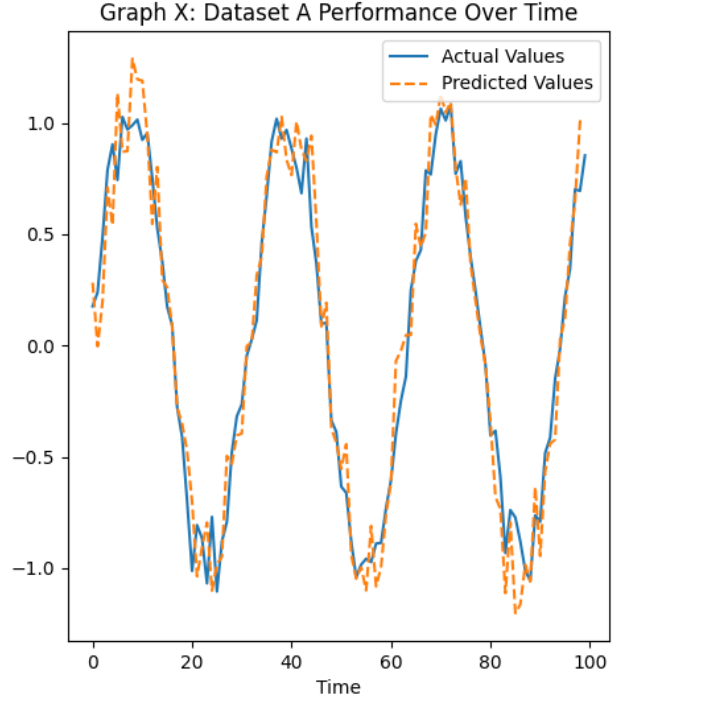


Graph X (Underground Drilling Machines Data - Dataset A)


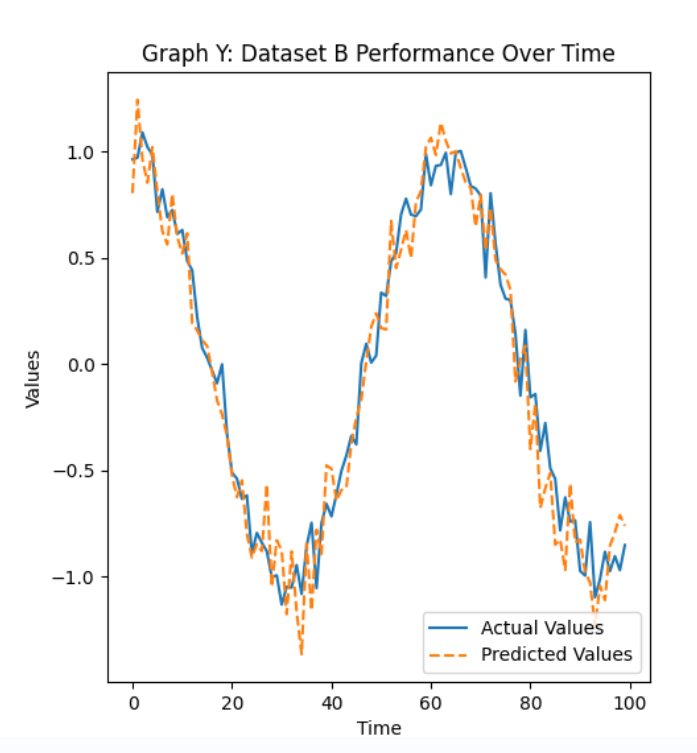


Graph Y (Green Stormwater Infrastructure Data - Dataset B)


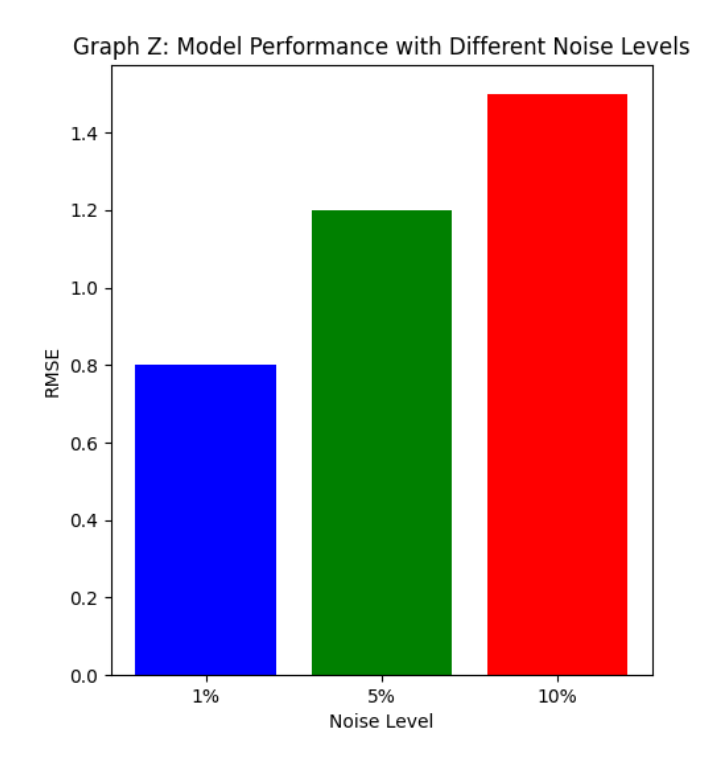


Graph Z (Model Performance with Different Noise Levels)
