# Supplementary material for: Advanced hybrid LSTM-transformer architecture for real-time multi-task prediction in engineering systems
Source: Sci Rep. 2024 Feb 28;14:4890. doi: 10.1038/s41598-024-55483-x (PMC11322354; doi:10.1038/s41598-024-55483-x)
Supplement: Supplementary file 1 — Supplementary Information. [file 41598_2024_55483_MOESM1_ESM.zip › Highlights.docx]

Highlights

1.Introduction of a novel hybrid LSTM-Transformer architecture for real-time predictions in complex engineering systems.

2.Integration of online learning, knowledge distillation, and adaptive mechanisms to ensure robustness and adaptability.

3.Rigorous ablation analysis to dissect the contributions of each architectural component.

4.Extensive experimental validation, backed by statistical measures, to confirm the model's reliability and efficiency.

5.Potential for immediate impact in critical engineering domains, particularly in underground drilling and green stormwater management.
