# Supplementary figures and images for: Advanced hybrid LSTM-transformer architecture for real-time multi-task prediction in engineering systems
Source: Sci Rep. 2024 Feb 28;14:4890. doi: 10.1038/s41598-024-55483-x (PMC11322354; doi:10.1038/s41598-024-55483-x)

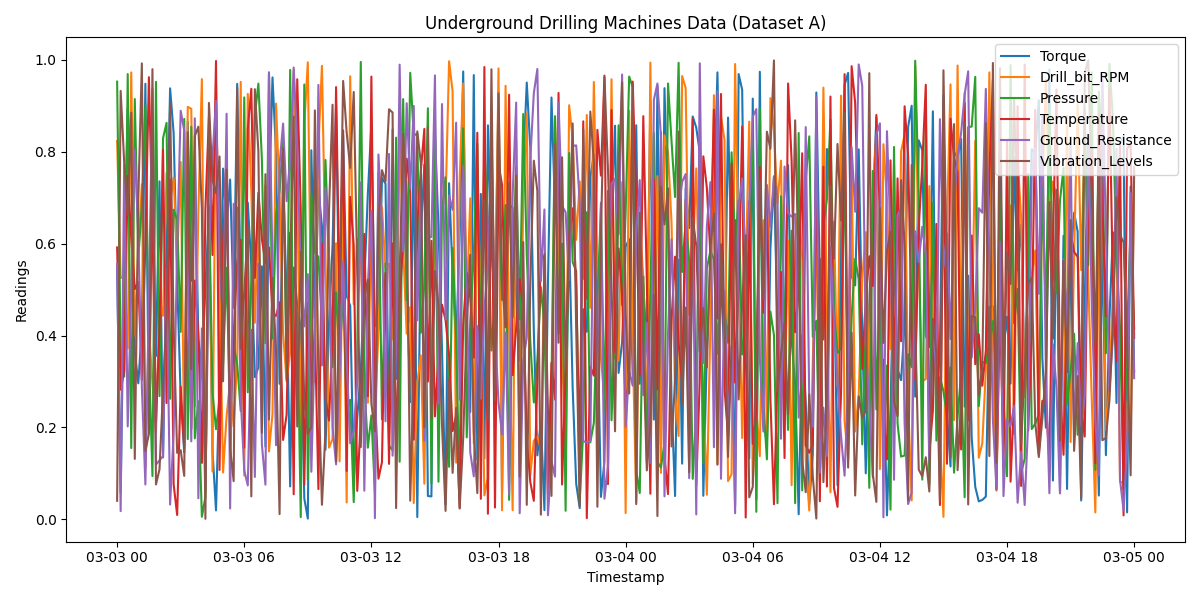

Supplement: Supplementary file 1 — Supplementary Information. [file 41598_2024_55483_MOESM1_ESM.zip › figure3.png]

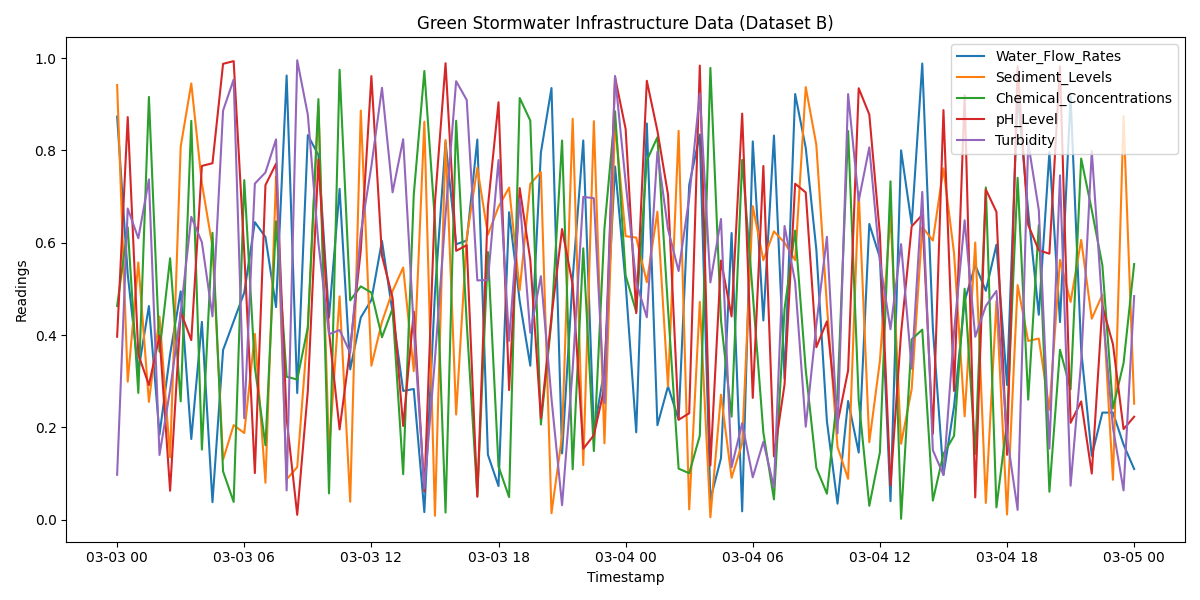

Supplement: Supplementary file 1 — Supplementary Information. [file 41598_2024_55483_MOESM1_ESM.zip › figure4.png]

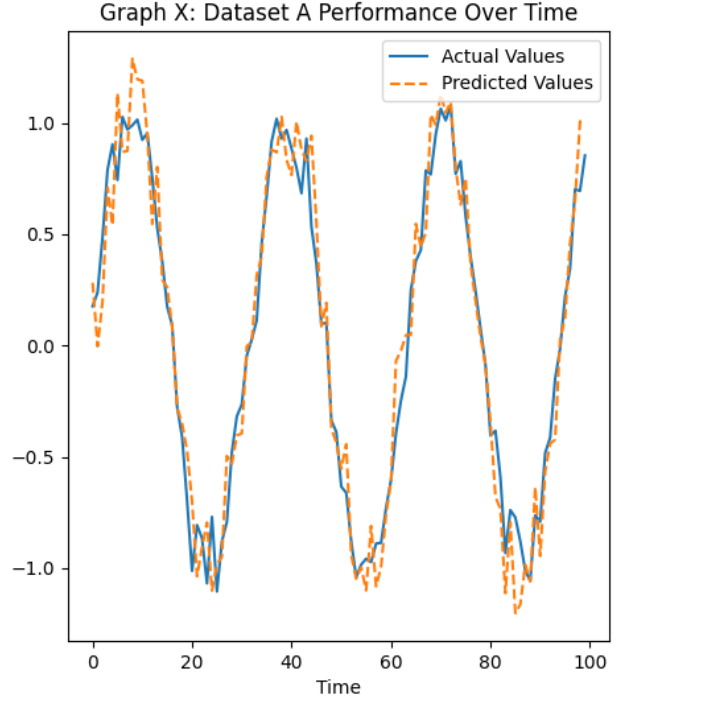

Supplement: Supplementary file 1 — Supplementary Information. [file 41598_2024_55483_MOESM1_ESM.zip › Gragh X.png]

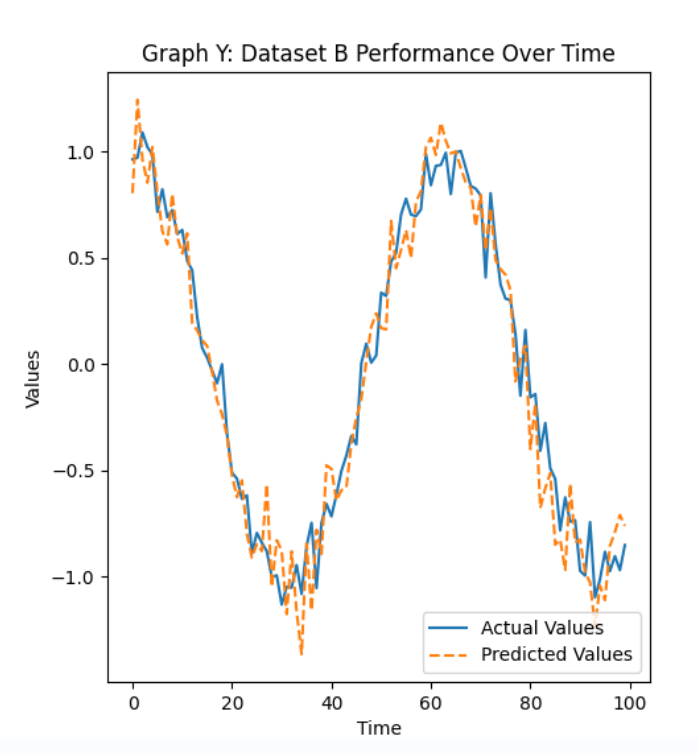

Supplement: Supplementary file 1 — Supplementary Information. [file 41598_2024_55483_MOESM1_ESM.zip › Gragh Y.png]

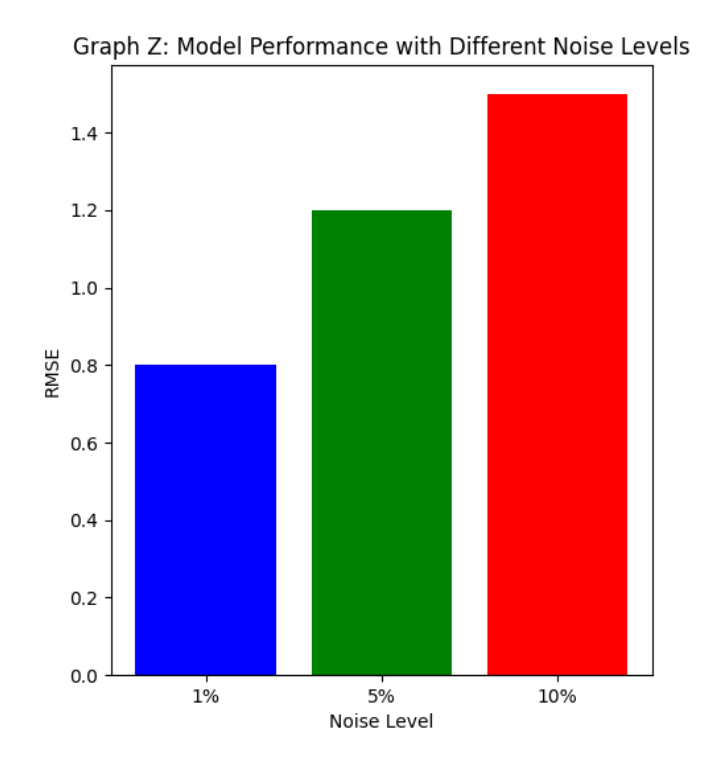

Supplement: Supplementary file 1 — Supplementary Information. [file 41598_2024_55483_MOESM1_ESM.zip › Gragh Z.png]
